# Comparative Evaluation of Conventional and Emerging Maceration Techniques for Enhancing Bioactive Compounds in Aronia Juice

**DOI:** 10.3390/foods13203255

**Published:** 2024-10-13

**Authors:** Alema Puzovic, Maja Mikulic-Petkovsek

**Affiliations:** Agronomy Department, Biotechnical Faculty, University of Ljubljana, 1000 Ljubljana, Slovenia; maja.mikulic-petkovsek@bf.uni-lj.si

**Keywords:** *Aronia melanocarpa*, maceration, ultrasound, microwave, extraction, sugars, acids, phenolic compounds

## Abstract

Ultrasound and microwave maceration techniques have been utilised to lower production costs and reduce processing time, while also preventing the degradation of nutrients like phenolics and vitamin C and preserving physical properties such as colour and viscosity. In this study, the effects of several traditional (cold, enzymatic, and thermal) and innovative (ultrasonic and microwave) maceration methods on some quality parameters of aronia juice were investigated. Microwave maceration significantly impacted the soluble solids content of the analysed juices and resulted in noticeably darker juice samples compared to the controls, with lower L*/lightness (20.1) and b*/blue-yellowness (−3.2) values and an increased a*/redness value (1.7). Different maceration methods also significantly impacted the rheological properties of the treated juices, among which MW treatment consistently showed a higher viscosity. Sorbitol and fructose were the main sugars identified, while malic acid and quinic acid accounted for 85% of the total acid content. Significant increases in the total sugar and acid concentrations were obtained in the juice samples from ultrasonic, microwave, and enzymatic maceration, while thermomaceration had no significant effect. The concentration of total phenolics ranged from 6.45 g/L in the thermomaceration samples to 9.86 and 14.07 g/L in the ultrasonic and microwave samples, respectively. The obtained results suggest that ultrasonic and microwave technologies were superior in terms of colour improvement and the extraction of sugars, acids, and phenolic compounds compared to traditional maceration methods. Ultrasound and microwave technologies present possible approaches to the improvement of aronia juice production in comparison to traditional methods.

## 1. Introduction

Aronia berries are rarely consumed fresh because of their unpleasant sensory attributes, such as bitterness and astringency, and they are used instead for the production of jams, juices, wines, and anthocyanin colorants [1,2]. Numerous health-promoting properties, such as anti-inflammatory, anticancer, antimicrobial, antiviral, antidiabetic, antiatherosclerotic, hypotensive, antiplatelet, and anti-inflammatory effects, have been related to the consumption of a polyphenol-rich diet, such as aronia berries and their products [3]. Therefore, due to its very high and versatile content of phenolic compounds, including procyanidins, anthocyanins, phenolic acids, and their analogues, aronia presents a valuable raw material for juice production [1,4]. However, several studies have shown that the content of phenolic compounds in fruit juices (FJs) strongly depends on how these FJs are produced, processed, and preserved [5,6]. For example, the pre-treatment of fruit mash can lead to different degrees of extraction of bioactive compounds, which changes the phytochemical composition of the FJ [7,8]. More traditional processing methods of FJ include thermomaceration and enzymatic maceration, especially with pectolytic enzymes, while novel strategies include the application of various methods such as microwave and ultrasound. Recently, the application of ultrasound and microwave for maceration has been explored, not only to reduce production costs and shorten processing time, but also to prevent the degradation of nutrients, including phenolics and vitamin C, and to preserve physical properties such as colour during juice processing [9]. Traditional maceration techniques, including cold, thermal, and enzymatic methods, are considered as low-cost and adaptable techniques, since they can be performed with simple-to-use equipment [10]. However, they have several disadvantages such as long extraction times, a high solvent consumption, a low yield, and a low efficiency [11]. Additionally, these techniques often lack selectivity in extraction, therefore, novel methods have been explored as an alternative to overcome these disadvantages, including microwave and ultrasound, among others [12]. The main advantages of microwave application for the extraction of bioactive compounds are related to the acceleration of the extraction process through uniform and rapid volumetric heating, the reduction in solvent consumption, and the improvement of matrix deconstruction, which promotes the release of larger amounts of compounds [13,14]. In comparison to traditional methods such as thermomaceration, in which heat is transferred by convection and conduction, the microwave process generates heat within the treated material, which increases the extraction rates and efficiency [11]. Ultrasound, on the other hand, induces the cavitation effect, which occurs at ultrasound frequencies between 20 and 100 kHz, forming gas bubbles in the treated material [5]. When these gas bubbles implode, they generate physical effects (such as microstreaming, microjets, and shock waves) and chemical reactions that disrupt the cell wall, facilitating the release of bioactive compounds [15]. Due to these characteristics, ultrasound and microwave technologies present a possible approach to the improvement of berry juice production, considering that they are more environmentally friendly and use less energy and less solvent while producing higher yields [15,16]. However, both technologies require further optimisation of their extraction parameters to achieve the best possible extraction outcomes based on the target bioactive components and substrate characteristics, which demands extensive experimentation [17].

The use of innovative technologies as an alternative to conventional extraction and maceration processes has been reported for various FJs such as grape, strawberry, and tangerine [9,18,19]. In the case of aronia, most studies focus on the use of US and MW for extraction from aronia berries [20,21,22,23], dried aronia berries [24], and aronia berry by-products [25,26]. Nemetz et al. (2023) [16] reported a positive effect of combined ultrasound-assisted enzymatic maceration on the composition of aronia juice, however, to our knowledge there has been no study investigating the effects of cold, thermal, enzymatic, ultrasonic, and microwave maceration techniques for processing aronia juice. Therefore, the objective of this research is to investigate the impact of cold, thermal, enzymatic, ultrasonic, and microwave technologies as maceration methods on the colour and rheological properties of aronia juice, as well as their effects on the extraction of sugars, organic acids, and phenolic compounds.

## 2. Materials and Methods

### 2.1. Mash Maceration

Aronia berries (*Aronia melanocarpa* L. cv. ‘Nero’) were obtained from a farm located in Novo Mesto (Slovenia) in July 2024 and stored at −18 °C until processing. The frozen berries were thawed before processing and milled to obtain a mash, which was macerated. Approximately 500 g of aronia berries was used per repetition for each maceration method. Prior to juice extraction, the following five different maceration techniques were applied:

- Cold maceration (CM)—the mash was stored at 4 °C for 12 h, slightly modifying the method described in Vagiri and Jensen (2017) [27]. Cold maceration is commonly used in processes like red winemaking, as it has practical benefits such as ease of implementation and low installation and maintenance costs [28]. Additionally, in this process, heat-sensitive compounds in the treated matrix remain largely unaltered due to the absence of heat [29]. Therefore, cold maceration was chosen as the control treatment because of its non-invasive nature and minimal processing impact on the properties of the aronia berries under study.
- Thermomaceration (TM) was carried out by heating the mash at 50 °C for 60 min, according to the method described in Lima et al. (2015) [30].
- Enzymatic maceration (EM) was performed by heating the mash until its internal temperature reached 50 °C, then pectolytic enzymes (ROHAPECT MC^®^) were added to achieve a concentration of 200 ppm with a holding time of 60 min at 50 °C, as described in Lima et al. (2015) [30]. The enzyme dosage, maceration time, and temperature were applied according to the manufacturers’ recommendations.
- Ultrasound-assisted maceration (US) was performed in an ultrasonic bath with a 250 W power and 37 kHz frequency at 60 °C for 15 min, as described in Lieu and Le (2010) [31].
- Microwave-assisted maceration (MW) was carried out according to the method described in Guler (2023) [9] by heating the fruit mash at a 600 W power for three cycles, with 2 min per cycle.

After maceration, the juice was pressed using a Hydro PARA-Press stainless-steel hydraulic press (part no. 0423, Paul Arauner GmbH & Co. KG, Kitzingen, Germany) under a pressure of 3.5 bar for 15 min and pasteurised in water bath at 85 °C for 1 min.

### 2.2. Determination of pH and Soluble Solids

The soluble solids (SS) content was determined with a Milwaukee MA885 digital refractometer (Milwaukee Instruments, Rocky Mount, NC, USA) and is expressed as °Brix. pH was determined potentiometrically at room temperature using a pH meter Mettler Toledo SevenEasy S20 (Mettler-Toledo AG., Schwerzenbach, Switzerland).

### 2.3. Rheology

The apparent viscosity of the aronia juice was determined using an Anton Paar rotational rheometer (ViscoQC 300 L, Anton Paar, Graz, Austria) equipped with a stainless-steel 26 mm measuring cup (C-DG26) and measuring bob (B-DG26). The samples were subjected to increasing shear rates from 1 s^−1^ to 250 s^−1^ at a constant temperature of 20 °C. The analysis was performed in triplicate, and the obtained data were recorded using the Anton Paar V-Collect PC data collection software (version no. 1.30.9109.34).

The variations in the shear rate and shear stress obtained from the measurements were used to analyse the rheological properties of the samples. The Ostwald–de Waele model, commonly applied to describe the flow behaviour of complex fluids like melts and polymer solutions, is widely used in processing applications due to its simplicity and the ease with which rheological parameters can be derived from the flow curve. The power-law model is expressed with the following equation:

$$\mu \left(\dot{\gamma }\right)=K{\gamma \;˙}^{n-1}$$

where *K* represents the viscosity at a shear rate of 1 s^−1^ and *n* is the power-law index, which indicates the rate of shear thinning when *n* < 1.

### 2.4. Colour Measurements

A colorimeter (CR-10 Chroma, Minolta, Osaka, Japan) was used to measure colour of the juices, expressed as L* (lightness), a* (redness), and b* (yellowness) values. The total colour difference *(*∆E*) of samples was calculated according to the following equation:

$$∆E^{*}=\sqrt{{\left(∆L*\right)}^{2}+{\left(∆a*\right)}^{2}+{\left(∆b*\right)}^{2}}$$

where ∆L*, ∆a*, and ∆b* represent the differences between the juice samples produced using different maceration techniques and the control sample. ΔE* represents the total colour difference between each treatment sample and the control group.

### 2.5. Determination and Quantification of Sugars and Organic Acids

The extraction and quantification of sugars and organic acids were conducted following the methodology by Mikulic-Petkovsek et al. (2012) [32]. The juice samples were centrifuged (Eppendorf Centrifuge 5810 R, Hamburg, Germany) for 5 min at 4 °C and 6000 rpm. After centrifugation, the juice was filtered through 0.20 µm cellulose filters (Macherey-Nagel, Düren, Germany) into glass vials and subjected to analysis using high-performance liquid chromatography (HPLC Vanquish ™ Flex UHPLC, Thermo Fisher Scientific, San Jose, CA, USA).

A Rezex RCM-monosaccharide Ca+ (2%) column (150 × 7.8 mm) (Phenomenex, Torrance, CA, USA) with bi-distilled water as the mobile phase was used to analyse the individual sugars. The parameters included a flow rate of 0.8 mL/min, a column temperature of 80 °C, and a total run time of 20 min. Organic acids were analysed using a Rezex ROA—organic acid H+ (8%) column (150 × 7.8 mm) (Phenomenex, Torrance, CA, USA) with a mobile phase of 4 mM sulfuric acid. For the organic acids, a single sample run lasted 15 min, with a flow rate of 0.6 mL/min and a column working temperature of 65 °C. A refractive index detector was used for the identification and measurement of sugars, while a UV detector at 210 nm was used for the analysis of organic acids. The concentrations of each metabolite were calculated based on the calibration curves of the corresponding standards and are expressed in g/L juice.

### 2.6. Determination and Quantification of Phenolic Compounds

An HPLC system (HPLC Finnigan Surveyor, Thermo Fischer Scientific, San Jose, CA, USA) equipped with a photodiode array detector (PDA) operating at three wavelengths (280, 350, and 530 nm) coupled with a mass spectrometer (MS) was used for the identification and quantification of phenolic compounds. The mobile phases comprised bi-distilled water/acetonitrile/formic acid (96.9/3/0.1, *v*/*v*/*v*) for mobile phase A and acetonitrile/bi-distilled water/formic acid (96.9/3/0.1, *v*/*v*/*v*) for mobile phase B. The elution process followed a linear gradient from 5% to 20% B in the initial 15 min, followed by a linear gradient from 20% to 30% B for 5 min, an isocratic phase for 5 min, another linear gradient from 30% to 90% B for 5 min, and a concluding isocratic phase for 15 min before reverting to the initial conditions [33]. A Gemini C18 column (Phenomenex, Torrance, CA, USA), maintained at 25 °C, was used. Positive ionisation modes were used for anthocyanins, while negative ionisation modes were applied for other phenolic compounds, employing full scan data-dependent MSn scanning from *m*/*z* 115 to 1900. The source parameters included a capillary temperature of 250 °C, sheath gas and auxiliary gas at 60 and 15 units, a source voltage of 3 kV, and normalised collision energy ranging from 20% to 35%. Data interpretation was performed using the Thermo Scientific™ Xcalibur™ 4.7 software (Thermo Scientific, Waltham, MA, USA). Phenolic compounds were identified based on retention times and PDA spectra, compared with phenolic standards, fragmentation patterns in various MSn modes, and data from the literature. The content of individual phenolic compounds was determined using standard curves for different phenolics, generated by injecting five concentrations of each phenolic compound three times. The concentration of the total phenolic compounds is expressed in g/L, and in mg/L for individual phenolic compounds.

### 2.7. Analysis of Ascorbic Acid Content

For the ascorbic acid (AA) analysis, a slightly modified method previously described in Mikulic-Petkovsek et al. (2013) [34] was used. Briefly, the juice samples were centrifuged for 5 min at 4 °C and 6000 rpm (Eppendorf centrifuge 5810R, Hamburg, Germany) and filtered into vials using a 0.20 µm cellulose mixed esters filter (Macherey-Nagel, Düren, Germany). The samples were analysed using a high-performance liquid chromatography (HPLC; Thermo Scientific, Diode Array Detector CG, Waltham, MA, USA) system coupled with a 245 nm wavelength UV detector for identification. A Rezex ROA-organic acid H+ (8%) column (300 mm × 7.8 mm) from Phenomenex (Torrance, CA, USA) was used for the separation of AA, with the column temperature set at 20 °C. The eluent was 4 mM sulphuric acid in bi-distilled water at a flow rate of 0.6 mL/min. The analysis duration was 30 min. The ascorbic acid concentrations were calculated using standard curves and are expressed as g/L of the ascorbic acid in the juice.

### 2.8. Statistical Analysis

The pH, SS, and viscosity measurements were performed in triplicate, while the rest of analytes (ascorbic acid, sugars, organic acids, and phenolics), as well as the colour measurements, were performed in four repetitions. The results are presented as mean values with standard error (mean ± SE). The differences between treatments were analysed by one-way ANOVA with Tukey’s test for significance (*p* ≤ 0.05), using the statistical program R-commander version 4.3.0 (R Formation for Statistical Computing, Auckland, New Zealand).

## 3. Results

### 3.1. PH and Soluble Solids

Some of the general physicochemical properties of the analysed aronia juices examined are presented in Table 1. The pH of the juices ranged between 3.47 and 3.48, with no significant differences between treatments. The SS values were determined from 14.9 to 17.93 °Brix for CM and MW maceration, respectively.

Statistical analysis showed significant differences between some of the treatments for SS. The samples obtained from MW and US maceration had the highest content of SS, with mean values of 17.93 and 16.17, respectively. However, only the MW maceration treatment resulted in a significant increase in SS compared to the CM, EM, and TM treatments. The US treatment showed no significant difference in SS values compared to the CM, EM, and TM treatments, as well as compared to the MW treatment. The pH and SS values were consistent with data from other authors [35,36], but depended on various factors, including the growing conditions of the aronia (e.g., soil, amount of precipitation, number of hours of sunlight, nutrients, and technology production), harvest time and variety, and pretreatment methods during juice processing [35,37]. Additionally, sugar content is a major contributor to the SS content in fruit juices, with sugar levels showing a positive correlation with SS [38,39]. Therefore, the increase in SS values observed in our results following the MW treatment could be attributed to the more efficient extraction of sugars observed in the MW samples. In a study by Brodie et al. (2011), the microwave treatment of cane prior to diffusion led to significant increases in Brix % as a result of cell damage [40]. The thermal effect of microwaves generates internal micro-fractures in cells, leading to a faster release of intracellular materials, including sugars and pectin [40,41]. Microwave treatment is, therefore, considered to be a green technology that enhances the extraction of soluble compounds by disrupting cellular structures through heating [42].

### 3.2. Rheological Behaviour

**Figure 1:**
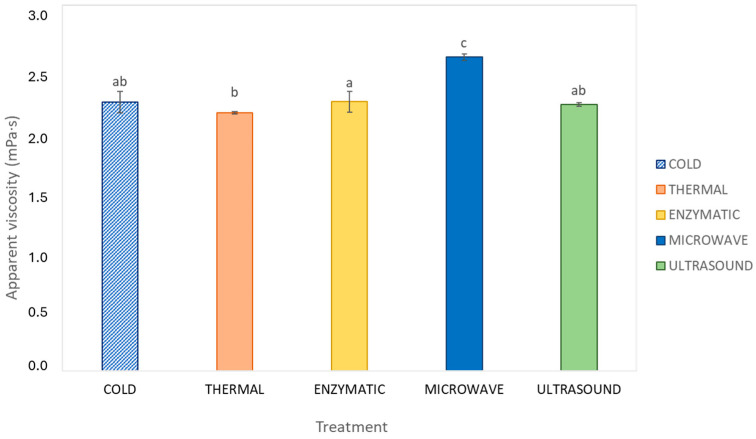
Apparent viscosity of aronia juices obtained with different treatments. The values are the means of three repetitions. Mean values followed by a different letter in the column are significantly different according to Tukey’s test (*p* ≤ 0.05).

The apparent viscosity (mPa·s) of the juice samples treated with different maceration methods was evaluated, and the results are presented in Figure 1. Statistical analysis showed significant differences in the viscosity between treatments (*p* ≤ 0.05). The samples obtained from the MW treatment had the highest apparent viscosity at 2.57 mPa·s, significantly higher than the rest of the treatments. In contrast, the TM treatment resulted in the lowest viscosity at 2.11 mPa·s, significantly lower than the MW and EM samples, however, this treatment was similar to CM and US. The apparent viscosity of the EM samples was significantly different and higher compared to the TM samples, but not as viscous as the samples obtained from the MW treatment. This indicates that the MW treatment produced juices with the highest viscosity across different shear rates, while the TM, CM, and US juices had relatively lower viscosities under similar conditions.

Fruit juices comprise pulp (insoluble phase) dispersed in a viscous solution (i.e., the serum), and their rheological behaviour is influenced by their composition, especially the type of fruit and the treatment performed in its processing. Maran et al. (2013) reported that the extraction efficiency of pectin could be improved by raising the MW power from 160 W to 480 W under the same solid–liquid ratio, caused by the direct effects of the MW energy on the plant materials [43]. More electromagnetic energy by increasing the MW power was transferred onto biomolecules by ionic conduction and dipole rotations, which resulted in more power dissipated inside the solvent and plant material and then quickly generated molecular movement and heating on the traction system, improving the pectin extraction efficiency [43]. Therefore, by causing disruption of the cell wall in samples during treatment, along with increasing the soluble pectin, a higher intensity of MW treatment can result in a product with a higher viscosity.

**Figure 2:**
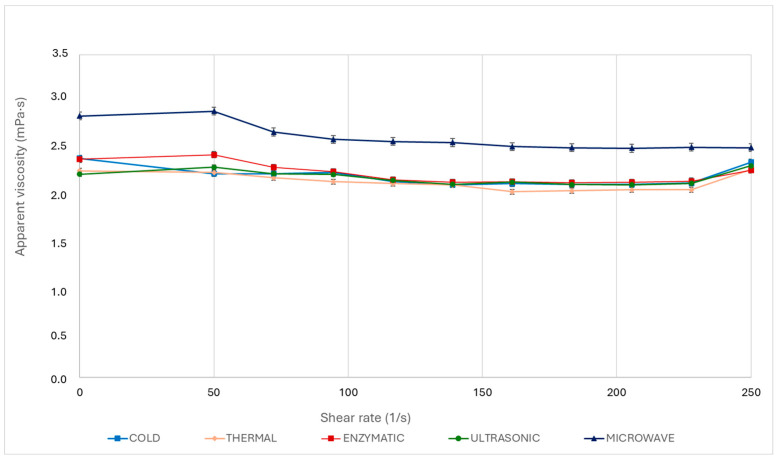
Viscosity curves of aronia juices across shear rate measured at 20 °C.

A higher dynamic viscosity at different shear rates typically suggests a better stability and a stronger interaction between particles in the fluid. Figure 2 shows the apparent viscosity (in mPa·s) plotted against the shear rate (in 1/s). Across the treatments, the apparent viscosity tended to decrease slightly as the shear rate increased. This was especially noticeable for shear rates from 0 to around 150 1/s, indicating the pseudoplastic or shear thinning behaviour of the examined juices. Pseudoplastic behaviour is a typical feature of many liquid food products like juices, in which viscosity decreases with an increasing shear rate.

Further insight into the values of the flow index showed all samples to display shear thinning behaviour, considering that the values of ‘n’ for all treatments were less than 1 (Table 2). These results confirm the typical pseudoplastic behaviour of treated aronia juices. On the other hand, higher ‘k’ values were associated with samples possessing a greater viscosity, with the highest value of 0.0038 observed for the MW treatment, in comparison to the 0.0023 value observed for the CM treatment.

These findings suggest that the different maceration methods significantly impacted the rheological properties of the treated juices, among which the MW treatment consistently showed a higher viscosity and shear stress. Understanding these rheological changes is essential for optimising processing parameters and achieving the desired consistency and quality of aronia juice.

### 3.3. Colour

The colour properties of the aronia juices are presented in Table 3 regarding the L*, a*, b*, and ΔE* values. The samples obtained from the CM, EM, and TM treatments did not show significant differences in their L* values, meaning that the lightness of the juices prepared by these methods was not affected. In contrast, the MW (20.13 ± 0.15) and US (20.28 ± 0.29) treatments had significantly lower L* values compared to the other treatments, meaning that the juices obtained with these treatments were darker in colour. Similarly, the CM, EM, and TM treatments were not significantly different from one another in terms of their a* values, showing similar redness levels. The juices obtained from the MW treatment (1.70 ± 0.03) had a significantly higher a* value, while the US treatment was not significantly different from the CM, EM, and TM treatments, but also not significantly different from the MW treatment. Additionally, statistical analysis indicated no significant differences in the b* values across the treatments, meaning that the treated juices exhibited a similar blue hue. Based on the ΔE* value, differences in perceivable colour are classified as not noticeable (0–0.5), slightly noticeable (0.5–1.5), noticeable (1.5–3.0), well visible (3.0–6.0), and great (6.0–12.0) [1]. Our results indicate that the microwave treatment had the greatest impact on juice colour, with ΔE* values of 1.59, resulting in samples that were significantly darker and contained more red and blue pigments compared to the CM samples. However, the differences in the ΔE* values between the MW, US, TM, and EM treatments were not significant (Table 3).

Fruit juice colour plays a crucial role in both its quality and appeal, and it is influenced by the treatments applied during processing. The production of juices requires the disintegration of fruit cells to release liquid, and thermal treatment of the raw material prior to pressing can further enhance anthocyanin extraction by breaking down tissues more effectively. As the skin anthocyanin content increases, the lightness of the juice decreases, resulting in a darker appearance. This correlation between anthocyanin concentration and juice darkness has been observed in various studies. Al Bittar et al., for instance, reported an increase in darkness for innovative grape juice produced by microwave-assisted extraction, where the juice, enriched with polyphenols, was darker and redder than the natural juice [44]. As explained in Nguyen and Nguyen (2018), ultrasonic treatment has the potential to increase anthocyanin yield through the implosion of cavitation bubbles in the plant material [45]. This process generates significant heat, which aids the extraction process, consequently affecting the colour. Aronia juice typically exhibits a wine red to deep purple colour, depending on the variety, hence, the increased darkness in the juice colour was possibly caused by the enhanced anthocyanin extraction due to further tissue breakdown from the MW and US treatments.

### 3.4. Sugars and Organic Acids

Fruit juice quality is significantly determined by the content and ratio of soluble sugars and organic acids. As presented in Table 4, the total sugar concentration in the juice samples was measured between 85.65 g/L for CM and 142.39 g/L for MW, corresponding to the range previously reported in the literature for aronia juice [46]. Sorbitol and fructose were the predominant sugars, accounting for 72% of the total sugar content. Glucose was also present to a considerable extent (23.43–39.21 g/L). A similar sugar profile for aronia juice was previously reported by Jurendić and Ščetar (2021) and King and Bolling (2020) [3,47], with the exception of Oziembłowski et al. (2022) [48], who reported higher levels of glucose than sorbitol. In addition, a small amount of sucrose (1.19–1.66 g/L) was detected, which is consistent with the findings of Denev et al. (2018) [49], who reported traces of sucrose in aronia. Regarding the effects of the treatments, significant differences (*p* ≤ 0.05) were found in the concentrations of fructose, glucose, sorbitol, and total sugars between the US and MW samples compared to the CM samples. The juice samples obtained after US and MW maceration had 64% and 66% higher total sugar concentrations, respectively, than the CM samples. In addition, EM had a positive effect on the total sugar extraction, increasing the concentration by 46% compared to CM. Positive effects of ultrasonic, microwave, and enzymatic treatments on greater sugar extraction have already been reported for grapes [31], acai [50], apple [51], and lingonberry juice [52].

Microwave and ultrasonic treatments have been widely studied in the food industry for enhancing the extraction of components of interest from plant sources, such as sugars and other carbohydrates [51,53,54,55]. Microwaves provide the rapid and efficient extraction of carbohydrates, because they penetrate into substrates and generate heat from within, inducing cell wall destruction caused by the forced superheating of water molecules entrapped and continuous collisions within the matrix, thus enhancing the extraction efficiency of the components contained in the plant bodies [53,56]. The degradation of carbohydrates is important for controlling the yield and quality of extracted carbohydrates, because the hydrothermal reaction of carbohydrates is a sequential reaction of extraction, hydrolysis, and degradation [56].

An optimised method of microwave extraction provided a complete recovery of the sugar and inositol composition from different legume seeds and their corresponding pods in short times, with a significant reduction in solvent volumes, when compared to conventional thermal extraction [55]. In another study by Tsubaki et al. (2013), a comparison between induction and microwave heating for the degradation of five neutral monosaccharides (glucose, galactose, mannose, arabinose, and xylose) was studied [57]. The authors reported a 1.1- to 1.5-fold higher rate of the degradation of monosaccharides with induction heating, concluding that microwave-assisted extraction had an advantage in preventing the unexpected degradation of extracted polysaccharides and maintaining their native chemical structures [56]. According to Abid et al. (2014) [51], the increase in the contents of sugars during ultrasonic treatment might be attributed to the breakage of cell, which causes the extraction of the sugars from intracellular spaces to the liquid. Therefore, the higher sugar concentration in juices treated with MW and US maceration can be attributed to cell wall disruption, which promotes the rapid release of sugars while minimising their degradation, thus preserving their native structures and increasing the overall yield.

Organic acids play an important role in maintaining food quality by influencing its organoleptic properties, such as flavour, colour, and aroma, while also enhancing its functionality through health-promoting properties like antioxidant and antimicrobial activity [58]. Several acids, such as citric, fumaric, and malic, are used as acidulants or stabilisers because of their ability to impair microbial growth in food [58,59]. Additionally, they can inhibit the growth of pathogenic intestinal bacteria, therefore improving intestinal function and promoting the human body’s absorption of catechin [60]. By lowering the gastric pH, organic acids also accelerate the conversion of pepsinogen to pepsin, enhancing the absorption of proteins, starch amino acids, and minerals [59,60]. Therefore, the consumption of beverages containing organic acids, such as aronia juice, is beneficial for preserving the microbial balance in the gastrointestinal tract and improving the absorption rate of several nutrients. Aronia berries are a rich source of organic acids, which contribute to their high biological relevance [23]. In the analysed samples, seven organic acids were detected, namely malic, quinic, ascorbic, citric, shikimic, oxalic, and tartaric acid. Tasinov et al. (2022) [61] also reported the presence of succinic and fumaric acid in aronia juice, however, those acids were not identified in our samples. The average concentration of total acids in the analysed samples was between 11.64 and 21.31 g/L, which is similar to the concentrations previously reported by Sosnowska et al. (2016) [62] for aronia juice. Malic acid (8.39–13.20 g/L) and quinic acid (1.24–4.75 g/L) were the major acids identified in our juice samples, accounting for 85% of the total acid content. Additionally, a small amount of citric acid was also detected in the juice samples (1.17–2.12 g/L). Shikimic, oxalic, and tartaric acids were identified as minor components in our aronia juice.

Regarding the effects of the treatments, TM was the only maceration method that had no significant effect on the extraction of total organic acids compared to the controls. In contrast, MW and US maceration had a significant effect on the extraction of organic acids and increased the concentration of total acids by 1.6 and 1.8 times compared to the CM samples. In addition, MW maceration was the only treatment that had a positive impact on the ascorbic acid content by increasing its concentration compared to the control samples, while the other treatments had no effect (Table 5).

EM also improved the total acid extraction, which increased by 24% compared to the CM samples. This is in accordance with the results from Lima et al. (2015) [30] for grape juice, where EM treatment significantly improved the extraction of organic acids. Aronia berries are moderately rich in pectin, and during enzymatic mash maceration, the middle lamellas of the fruit are depolymerised by breaking pectin polymers into smaller fragments, i.e., monomer sugars, which improves the extraction of intracellular compounds [63,64,65]. Similar results of increasing the organic acid content by ultrasonic and microwave treatment were previously reported for various FJs [66,67,68]. However, Piecko et al. (2024) [15] reported no significant increase in the organic acid content of raspberry juice after the US pretreatment of the mash, which is in contrast to our results. According to Abid et al. (2014) [51], cell damage due to mechanical effects, exerted by shear forces during US treatment, improves the diffusion rate of intracellular contents from the material. The microwave effect is thermal; however, it was more efficient in the extraction of sugars and acids than the conventional heating used in TM. The reason for this is that microwaves heat the product directly and keep the temperature gradient to a minimum, which allows the microwaves to generate the heat within the sample/material and accelerate the rate of heating [69]. Conventional heating is based on heat conduction from external surfaces, which is why a certain amount of time is needed to transfer the heat to the sample/material [9,69]. This means that, during microwave treatment, cell wall disruption occurs because of the heat and pressure built up inside the plant cell and then transferred from inside the plant cell to the outside and distributed throughout the sample, allowing for better extraction [70].

### 3.5. Phenolic Compounds

**Figure 3:**
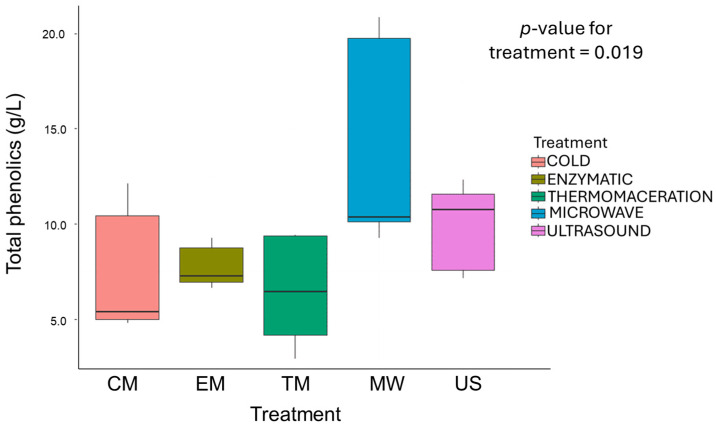
The effect of maceration methods on total phenolics (g/L). The values are the means of four repetitions.

Figure 3 summarises the concentrations of the total analysed phenolics quantified in the macerated samples. As presented, the concentration of total phenolics ranged from 6.45 g/L in the samples obtained from TM to 14.07 g/L in the MW samples. Compared to the literature, our results were in similar range to those reported by Tolić et al. (2015) [35] and Jakobek et al. (2007) [71] for aronia juice (6.64 g/L to 9.15 g/L), with the exception of the MW samples, which had higher values than the reported range. Both the phenolic profile and content of phenolic compounds in fruits are influenced by many factors, mostly genetic and environmental, but can be also modified during fruit processing [72]. For instance, fresh berries and pomace, a by-product of juice pressing, contain higher concentrations of phenolic compounds compared to juice, due to a greater amount of berry skin, which is abundant in phenolic compounds [73].

The aronia juice samples obtained by US and MW maceration showed significantly higher levels of total analysed phenolics than the CM samples, with increases of 30.7% and 86.5%, respectively. This could have been due to the effect of microwaves and ultrasound on the cell wall, which caused the release of phenolic compounds deposited in the cell vacuoles either in soluble or bound form. As explained in the previous section, microwave treatment allows for efficient extraction by rapidly heating the material/mash, while ultrasound causes either the disruption of cell walls or the formation of cavitation bubbles due to high pressure [74]. During the treatment, these bubbles expand to a critical size, become unstable, and collapse violently, allowing for improved phenolic extraction [75]. The vacuole is the most important organelle for the chemical quality of fruit, as it contains compounds responsible for the taste and flavours of fruits, such as sugars, organic acids, and secondary metabolites, all of which are found in the vacuole and can be present in very high concentrations [76].

Other authors also reported an improved extraction efficiency of anthocyanins and other phenolic compounds by MW and US treatments, and our results agree with those in the literature [9,31,77]. In contrast, TM and EM had no significant effect on the extraction rate of total phenolics compared to CM, which could be the result of the one-hour holding time used in these treatments. Thermomaceration and enzymatic maceration are usually carried out at a temperature of 50 °C, but the long maceration time is a disadvantage of these traditional methods, which reduces the content of extracted phenolics [78]. Polyphenols are widely recognised as unstable compounds, highly susceptible to degradation. The decrease in phenolic extraction during long maceration times can be attributed to several factors related to the stability of polyphenols under prolonged exposure to extraction conditions. Factors such as light, oxygen, and temperature can lead to the oxidative degradation of polyphenols, reducing the efficiency of their extraction [79,80].

During long maceration periods, partially extracted polyphenols are continuously exposed to air and light, increasing the likelihood of oxidative reactions that degrade these compounds [79]. High temperatures, e.g., those above 70 °C, further accelerate the degradation process, which was previously reported for anthocyanins [81]. This is why prolonged extraction processes, especially those involving higher temperatures, are associated with lower phenolic yields [79,82]. Prolonged maceration, while allowing for more time for diffusion, also prolongs exposure to conditions that facilitate the breakdown of these sensitive compounds, thereby reducing the phenolic content in the final extract. In contrast, novel extraction methods like microwave- or ultrasound-assisted extraction, offer faster extraction times and overcome the disadvantage of the long maceration time present in conventional methods [81,83]. Therefore, the observed reduction in phenolic extraction during extended maceration times is possibly caused by the oxidative degradation of phenolic compounds, which is not economically feasible for industrial applications [84].

**Figure 4:**
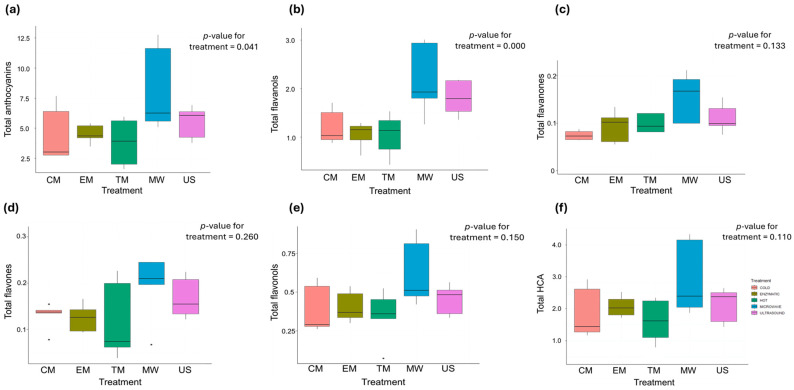
The effect of maceration methods on (**a**) total anthocyanins (g/L); (**b**) total flavanols (g/L); (**c**) total flavanones (g/L); (**d**) total flavones (g/L); (**e**) total flavonols (g/L); and (**f**) total HCA (g/L). The values are the means of four repetitions.

The phenolic compounds found in aronia can be divided into flavonoids (flavanols, anthocyanins, and flavonols) and non-flavonoids (phenolic acids) [85]. In our study, the following groups of phenolic compounds were identified in the aronia juice samples (Figure 4): anthocyanins, flavones, flavonols, flavanols, flavanones, and hydroxycinnamic acids (HCAs). Among the total phenolics, anthocyanins were the most abundant group, accounting for 58.2%, with an average concentration ranging from 3.82 to 8.26 g/L for the TM and MW treatments, respectively. The second most abundant group was the total HCAs, with an average concentration ranging from 1.54 to 2.96 g/L for the TM and MW treatments, respectively, accounting for 23% among the total phenolics. The total flavanols accounted for 13.5% among the total phenolics, with a concentration ranging from 0.715 to 2.19 g/L in the TM and MW samples, respectively. The total flavones, total flavonols, and total flavanones were present in small quantities, together accounting for 5.2% of the total phenolic content. Significant differences between the applied treatments were observed only for the total anthocyanins and total flavanols (Figure 4a,b). The juices obtained from MW maceration had the highest concentration of total anthocyanins in comparison to the TM juices, increasing their concentration twofold. In contrast, the TM samples had the lowest anthocyanin concentration, significantly lower than the MW samples, but not significantly different from the CM, EM, or US samples. Additionally, no significant differences were observed between the CM, EM, and US treatments in the total anthocyanin concentration. Similarly, the concentration of total flavanols significantly improved with the MW and US macerations by 2.5 and 3-fold, compared to the CM samples, while TM and EM maceration had no effect on the extraction of flavanols.

Aronia contains a high amount of phenolics compared to other berry species, such as blackberry, raspberry, and strawberry [86], which contributes to its high antioxidant activity and many potential benefits. Therefore, our study also analysed the individual phenolic compounds of aronia, which are listed in Table S2 in the Supplementary Materials. Flavanols comprised 14 individual compounds, mainly procyanidin derivatives. Catechin was quantified in the highest amount among the flavanols, with its concentration significantly increasing by 60% in both the US and MW samples, compared to CM. Among the twelve identified derivatives of hydroxycinnamic acids, caffeic acid and coumaric acid derivatives were the most represented in terms of quantity. Significant differences between the treatments were observed only for dicaffeoylquinic acid and 3-caffeoylquinic acid, for which MW maceration caused an increase in the concentration by 174% and 122% compared to the CM and TM samples, respectively. Flavonols mostly consisted of quercetin derivatives, however, no significant differences were observed between the treatments for any of the identified compounds. Anthocyanins were mainly cyanidin derivatives, with one pelargonidin hexose identified. As for the effects of the treatments, MW maceration significantly improved the concentration of both cyanidin-3-galactoside and cyanidin-3-glucoside twofold, in comparison to the TM treatment. Apigenin dirhamnoside was the only identified flavone, however, no significant differences were found in its concentration between the treatments. Similarly, two flavanones, namely naringenin hexoside 1 and naringenin hexoside 3, were identified in the analysed juices, but without significant differences between the treatments.

In general, the microwave and ultrasonic processing techniques were superior to the other maceration methods used in the extraction of both the total and individual phenolic compounds in aronia juice.

## 4. Conclusions

Different maceration methods significantly affected the colour, sugar, acid, and phenolic compound extraction of aronia juice. The ultrasonic and microwave treatments increased the total sugar concentration by 64 and 66%, respectively, and enhanced the extraction of organic acids by 61% and 83%, compared to cold maceration. Ultrasound and microwave were also superior in the extraction of phenolic compounds, with increases of 30.7% and 86.5% in their total content, respectively, compared to cold maceration. In contrast, thermomaceration and enzymatic maceration did not improve phenolic extraction, possibly due to the long holding time and lower extraction efficiency at 50 °C. These results suggest that ultrasound and microwave could be promising alternatives to traditional maceration methods, as they resulted in a better extraction efficiency of the analysed chemical compounds. However, the juices produced with microwave and ultrasonic treatments were darker than those produced with cold maceration, displaying the largest colour difference (∆E*) in comparison. Additionally, the MW treatment significantly increased the viscosity of the aronia juice, hence, further studies on the sensory impacts of ultrasound and microwave treatments on aronia juice would provide a more comprehensive understanding of the benefits and potential drawbacks of these methods, ensuring that the final product meets consumer expectations.

## Figures and Tables

**Table 1:** pH and soluble solids content of aronia juice prepared by different maceration methods.

Treatment	pH			SS		
	Mean	±SE	Sign.	Mean	±SE	Sign.
Cold	3.48	0.01	a	14.90	0.76	a
Enzymatic	3.48	0.01	a	15.90	0.17	a
Thermal	3.48	0.01	a	15.73	0.09	a
Microwave	3.47	0.01	a	17.93	0.13	b
Ultrasound	3.48	0.01	a	16.17	0.37	ab
*p* value			0.785			0.004

The values are the means of triplicate juice samples. Mean values followed by a different letter in the column are significantly different according to Tukey’s test (*p* ≤ 0.05).

**Table 2:** Consistency index (k) and flow index (n) of aronia juice samples obtained with different treatments.

Treatment	k			n			R^2^
	Mean	±SE	Sign.	Mean	±SE	Sign.	
Cold	0.0023	0.0001	a	0.9859	0.0150	b	0.9861
Enzymatic	0.0030	0.0001	a	0.9376	0.0046	b	0.9927
Thermal	0.0025	0.0001	a	0.9684	0.0066	b	0.9855
Microwave	0.0038	0.0001	b	0.9204	0.0096	a	0.9989
Ultrasound	0.0025	0.0001	a	0.9704	0.0094	b	0.9892
*p* value			0.000			0.005	

The values are the means of four repetitions. Mean values followed by a different letter in the column are significantly different according to Tukey’s test (*p* ≤ 0.05).

**Table 3:** Colour properties of aronia juices prepared by different maceration methods.

Treatment	L*			A*			B*			ΔE*		
	Mean	±SE	Sign.	Mean	±SE	Sign.	Mean	±SE	Sign.	Mean	±SE	Sign.
Cold	21.70	0.24	a	1.50	0.04	a	−3.10	0.04	a	0.00	0.00	a
Enzymatic	21.35	0.09	a	1.55	0.03	a	−3.30	0.15	a	0.71	0.09	ab
Thermal	21.15	0.13	a	1.45	0.03	a	−3.07	0.05	a	0.80	0.10	ab
Microwave	20.13	0.15	b	1.70	0.03	b	−3.20	0.07	a	1.59	0.09	b
Ultrasound	20.28	0.29	b	1.59	0.04	ab	−3.30	0.07	a	1.45	0.11	b
*p* value			0.000			0.001			0.227			0.000

The values are the means of four repetitions. Mean values followed by a different letter in the column are significantly different according to Tukey’s test (*p* ≤ 0.05).

**Table 4:** Content of total and individual sugars in aronia juices prepared by different maceration methods expressed in g/L.

Treatment	Total Sugars			Fructose			Glucose			Sorbitol			Sucrose		
	Mean	±SE	Sign.	Mean	±SE	Sign.	Mean	±SE	Sign.	Mean	±SE	Sign.	Mean	±SE	Sign.
Cold	85.65	0.37	a	28.66	0.21	a	23.43	0.60	a	32.37	0.27	a	1.19	0.05	a
Enzymatic	125.08	0.21	b	34.88	0.40	b	29.85	0.53	b	58.95	0.38	b	1.41	0.14	a
Thermal	87.48	0.92	a	29.53	0.49	a	23.69	0.57	a	33.01	0.31	a	1.25	0.06	a
Microwave	142.39	2.36	c	41.17	0.84	c	39.21	1.19	c	60.35	0.71	b	1.66	0.25	a
Ultrasound	140.19	2.19	c	40.57	0.78	c	37.65	1.26	c	60.40	0.25	b	1.57	0.17	a
*p* value			0.000			0.000			0.000			0.000			0.212

The values are the means of four repetitions. Mean values followed by a different letter in the column are significantly different according to Tukey’s test (*p* ≤ 0.05).

**Table 5:** Content of total and individual acids in aronia juices prepared by different maceration methods expressed in g/L.

Treatment	Total Acids			Citric			Malic			Oxalic			Quinic			Shikimic			Tartaric			Ascorbic		
	Mean	±SE	Sign.	Mean	±SE	Sign.	Mean	±SE	Sign.	Mean	±SE	Sign.	Mean	±SE	Sign.	Mean	±SE	Sign.	Mean	±SE	Sign.	Mean	±SE	Sign.
Cold	11.64	0.09	a	1.17	0.06	a	8.39	0.11	a	0.39	0.07	a	1.24	0.05	a	0.18	0.03	a	0.17	0.01	a	0.10	0.01	a
Enzymatic	14.49	0.24	b	1.49	0.15	ab	9.14	0.13	a	0.45	0.04	a	3.00	0.19	b	0.15	0.02	a	0.15	0.03	a	0.10	0.00	a
Thermal	11.92	0.15	a	1.14	0.08	a	8.63	0.21	a	0.36	0.04	a	1.35	0.14	a	0.16	0.02	a	0.19	0.01	a	0.11	0.00	a
Microwave	21.31	0.62	d	2.12	0.23	b	13.20	0.26	b	0.70	0.08	b	4.75	0.36	c	0.19	0.03	a	0.21	0.01	a	0.14	0.01	b
Ultrasound	18.77	0.59	c	1.94	0.17	b	12.22	0.36	b	0.46	0.04	ab	3.69	0.22	b	0.16	0.03	a	0.19	0.01	a	0.11	0.00	a
*p* value			0.000			0.001			0.000			0.005			0.000			0.727			0.307			0.003

The values are the means of four repetitions. Mean values followed by a different letter in the column are significantly different according to Tukey’s test (*p* ≤ 0.05).

## Data Availability

The original contributions presented in the study are included in the article/Supplementary Material, further inquiries can be directed to the corresponding author.

## Supplementary Materials

The following supporting information can be downloaded at: https://www.mdpi.com/article/10.3390/foods13203255/s1, Table S1: Concentration of total phenolics categorised by identified groups in the analysed juice samples (g/L); Table S2: The concentration of individual phenolic compounds in analysed juices (mg/L) prepared by different maceration methods; Figure S1: Photographs of the analysed juices prepared by different maceration methods; Figure S2: Chromatogram of detected individual sugars in aronia juice samples; Figure S3: Chromatogram of detected individual acids in aronia juice samples; Figure S4: Chromatogram of detected D-ascorbic acid and L-ascorbic acid; Figure S5: Chromatograms of detected individual phenolic compounds in aronia juice samples.
