# Supplementary material for: Comparative Evaluation of Conventional and Emerging Maceration Techniques for Enhancing Bioactive Compounds in Aronia Juice
Source: Foods. 2024 Oct 13;13(20):3255. doi: 10.3390/foods13203255 (PMC11507980; doi:10.3390/foods13203255)
Supplement: Supplementary file 1 [file foods-13-03255-s001.zip › foods-3209258-supplementary.pdf]

Table S1. Concentration of total phenolics categorized by identified groups in the analyzed juice samples (g/L).

Treatment	Total anthocyanins			Total flavones			Total flavonols			Total flavanols			Total flavanones			Total HCA			Total phenolics		
	mean	± SE	sign.	mean	± SE	sign.	mean	± SE	sign.	mean	± SE	sign.	mean	± SE	sign.	mean	± SE	sign.	mean	± SE	sign.
**COLD**	4.518	1.044	ab	0.013	0.013	a	0.401	0.788	a	0.727	0.063	a	0.008	0.005	a	1.878	3.663	a	7.545	15.498	ab
**ENZYMATIC**	3.818	0.350	ab	0.013	0.017	a	0.361	0.512	a	0.715	0.025	a	0.011	0.018	a	1.538	1.503	a	6.455	5.219	ab
**HOT**	4.524	0.892	a	0.013	0.043	a	0.413	0.851	a	0.748	0.111	a	0.010	0.036	a	2.069	3.632	a	7.777	13.221	a
**MICROWAVE**	8.264	1.624	b	0.021	0.041	a	0.624	0.977	a	2.186	0.340	b	0.017	0.034	a	2.956	5.324	a	14.067	25.602	b
**ULTRASOUND**	5.475	0.617	ab	0.017	0.020	a	0.450	0.439	a	1.803	0.164	b	0.011	0.016	a	2.108	2.482	a	9.863	10.592	b
p value			0.041			0.260			0.150			0.000			0.133			0.110			0.019

The values are the means of four repetitions. Mean values followed by a different letter in the column are significantly different according to Tukey's test ($p \leq 0.05$).

Table S2. The concentration of individual phenolic compounds in analyzed juices (mg/L) prepared by different maceration methods.

	COLD			THERMAL			ENZYMATIC			ULTRASOUND			MICROWAVE		
**Phenolic compound**	mean	±SE	sign.	mean	±SE	sign.	mean	±SE	sign.	mean	±SE	sign.	mean	±SE	sign.
ANTHOCYANINS															
**Cyanidin-3-galactoside**	3132.360	0.579	ab	2594.162	0.551	a	3042.195	0.180	ab	3767.965	0.359	ab	5798.061	0.952	b
**Cyanidin-3-glucoside**	211.516	0.035	a	185.595	0.027	a	233.475	0.016	ab	268.273	0.012	ab	394.534	0.051	b
**Cyanidin-3-arabinoside**	896.415	0.174	a	767.252	0.127	a	941.912	0.058	a	1079.127	0.101	a	1571.890	0.249	a
**Pelargonidin-3-galactoside**	7.884	0.002	a	6.748	0.001	a	8.284	0.001	a	9.491	0.001	a	13.825	0.002	a
**Cyanidin-3-xyloside**	243.143	0.057	a	236.129	0.032	a	263.633	0.035	a	309.866	0.030	a	434.993	0.066	a
**Cyanidin-3-O-hexosylpyruvate**	27.015	0.008	a	27.773	0.007	a	34.225	0.007	a	40.123	0.005	a	50.669	0.009	a
FLAVONES															
**Apigenin dirhamnoside**	12.931	0.001	a	12.597	0.004	a	12.931	0.001	a	16.838	0.002	a	20.722	0.003	a
FLAVONOLS															
**Quercetin dirhamnosylhexoside**	7.486	0.001	a	7.302	0.002	a	8.934	0.002	a	10.156	0.001	a	14.168	0.002	a
**Quercetin dihexoside 1**	36.264	0.006	a	37.209	0.007	a	39.303	0.005	a	41.977	0.004	a	57.233	0.007	a
**Quercetin dihexoside 2**	18.344	0.002	a	21.388	0.005	a	18.511	0.002	a	20.859	0.001	a	28.093	0.003	a
**Quercetin-3-vicianoside**	58.102	0.008	a	49.991	0.010	a	59.800	0.006	a	63.529	0.005	a	87.754	0.012	a

**Quercetin-3-robinobioside**	3.737	0.001	a	5.250	0.002	a	5.447	0.001	a	6.255	0.002	a	10.316	0.003	a
**Quercetin-3-rutinoside**	129.673	0.021	a	107.166	0.022	a	127.871	0.013	a	137.973	0.013	a	181.465	0.023	a
**Quercetin-3-galactoside**	84.878	0.017	a	75.973	0.014	a	89.340	0.009	a	98.272	0.010	a	142.893	0.022	a
**Isorhamnetin pentosylhexoside**	2.948	0.001	a	2.639	0.000	a	3.103	0.000	a	3.413	0.000	a	4.963	0.001	a
**Quercetin-3-glucoside**	59.740	0.010	a	53.763	0.010	a	60.889	0.005	a	67.625	0.006	a	96.759	0.013	a
FLAVANOLS															
**Procyanidin pentamer 1**	0.683	0.000	a	2.034	0.001	a	0.846	0.000	a	6.107	0.001	b	6.894	0.001	b
**Procyanidin pentamer 2**	0.053	0.000	a	0.050	0.000	a	0.066	0.000	a	4.309	0.001	b	3.728	0.001	b
**procyanidin hexamer 1**	2.939	0.000	a	2.492	0.000	a	3.493	0.000	a	44.541	0.005	b	45.057	0.004	b
**procyanidin dimer 1**	30.921	0.009	a	26.991	0.010	a	18.380	0.003	a	218.529	0.017	b	227.501	0.025	b
**catechin**	111.657	1.687	ab	828.738	1.440	a	842.676	0.991	a	1792.378	0.825	b	1775.727	1.578	b
**procyanidin hexamer 2**	71.657	0.471	a	728.738	1.145	a	742.676	0.632	a	1652.378	0.826	b	1955.727	0.123	b
**procyanidin dimer 2**	4.009	0.001	a	1.754	0.000	a	4.756	0.002	a	272.813	0.051	b	246.751	0.039	b
**procyanidin tetramer 1**	48.243	0.011	a	43.620	0.017	a	14.605	0.001	a	227.373	0.030	b	257.009	0.029	b
**epicatechin**	52.798	0.011	a	32.279	0.015	a	47.718	0.014	a	195.220	0.023	b	239.266	0.049	b
**procyanidin trimer 2**	1.490	0.000	a	2.434	0.001	a	1.930	0.000	a	16.145	0.002	ab	32.586	0.008	b
**procyanidin tetramer 2**	130.917	0.028	a	157.940	0.035	a	147.696	0.017	a	140.702	0.017	a	212.770	0.044	a

**procyanidin dimer 3**	102.642	0.022	a	108.950	0.022	a	115.142	0.010	a	103.043	0.011	a	170.966	0.039	a
**procyanidin tetramer 3**	105.240	0.024	a	89.047	0.010	a	126.982	0.023	a	96.684	0.012	a	178.603	0.039	a
**procyanidin tetramer 4**	63.388	0.012	a	92.105	0.022	a	108.282	0.017	a	133.056	0.020	a	191.321	0.046	a
FLAVANONES															
**Naringenin hexoside 1**	3.255	0.000	a	5.504	0.001	a	5.242	0.001	a	5.285	0.001	a	7.365	0.002	a
**Naringenin hexoside 2**	4.211	0.000	a	5.150	0.002	a	4.476	0.001	a	5.562	0.001	a	9.531	0.001	a
HYDROXYCYNNAMIC ACIDS															
**Dicaffeoylquinic acid**	1.513	0.000	a	2.054	0.001	ab	1.862	0.000	a	2.214	0.000	ab	4.147	0.001	b
**Caffeic acid**	51.081	0.072	a	43.319	0.061	a	33.077	0.088	a	43.102	0.084	a	68.552	0.086	a
**3-caffeoylquinic acid**	662.739	0.124	ab	522.262	0.134	a	823.274	0.069	ab	790.800	0.071	ab	1230.476	0.205	b
**caffeic acid hexoside 1**	7.127	0.001	a	5.825	0.001	a	7.483	0.001	a	7.251	0.001	a	10.079	0.002	a
***p*-coumaric acid hexoside**	28.417	0.004	a	23.225	0.004	a	29.836	0.003	a	28.912	0.003	a	40.187	0.006	a
**3-*p*-coumaroylquinic acid**	33.726	0.006	a	29.921	0.006	a	32.636	0.003	a	36.419	0.005	a	52.937	0.006	a
**5-caffeoylquinic acid 1**	1016.568	1.450	a	828.738	1.440	a	1042.676	1.115	a	1112.378	1.203	a	1415.727	1.867	a
**5-Caffeoylquinic acid 2**	37.752	0.006	a	39.204	0.006	a	51.769	0.006	a	44.319	0.004	a	65.837	0.011	a
**4-Feruloylquinic acid**	8.706	0.002	a	8.731	0.001	a	11.145	0.001	a	10.658	0.001	a	14.702	0.002	a
**5-*p*-coumaroylquinic acid 1**	6.362	0.002	a	10.393	0.004	a	8.240	0.001	a	6.893	0.001	a	13.570	0.003	a

**5-feruloylquinic acid**	14.155	0.002	a	14.940	0.003	a	14.862	0.001	a	14.337	0.001	a	24.169	0.006	a
**5-*p*-coumaroylquinic acid 2**	9.934	0.002	a	9.316	0.001	a	12.108	0.001	a	10.321	0.001	a	15.810	0.003	a

The values are the means of four repetitions. Mean values followed by a different letter in the row are significantly different according to Tukey's test ($p \leq 0.05$).

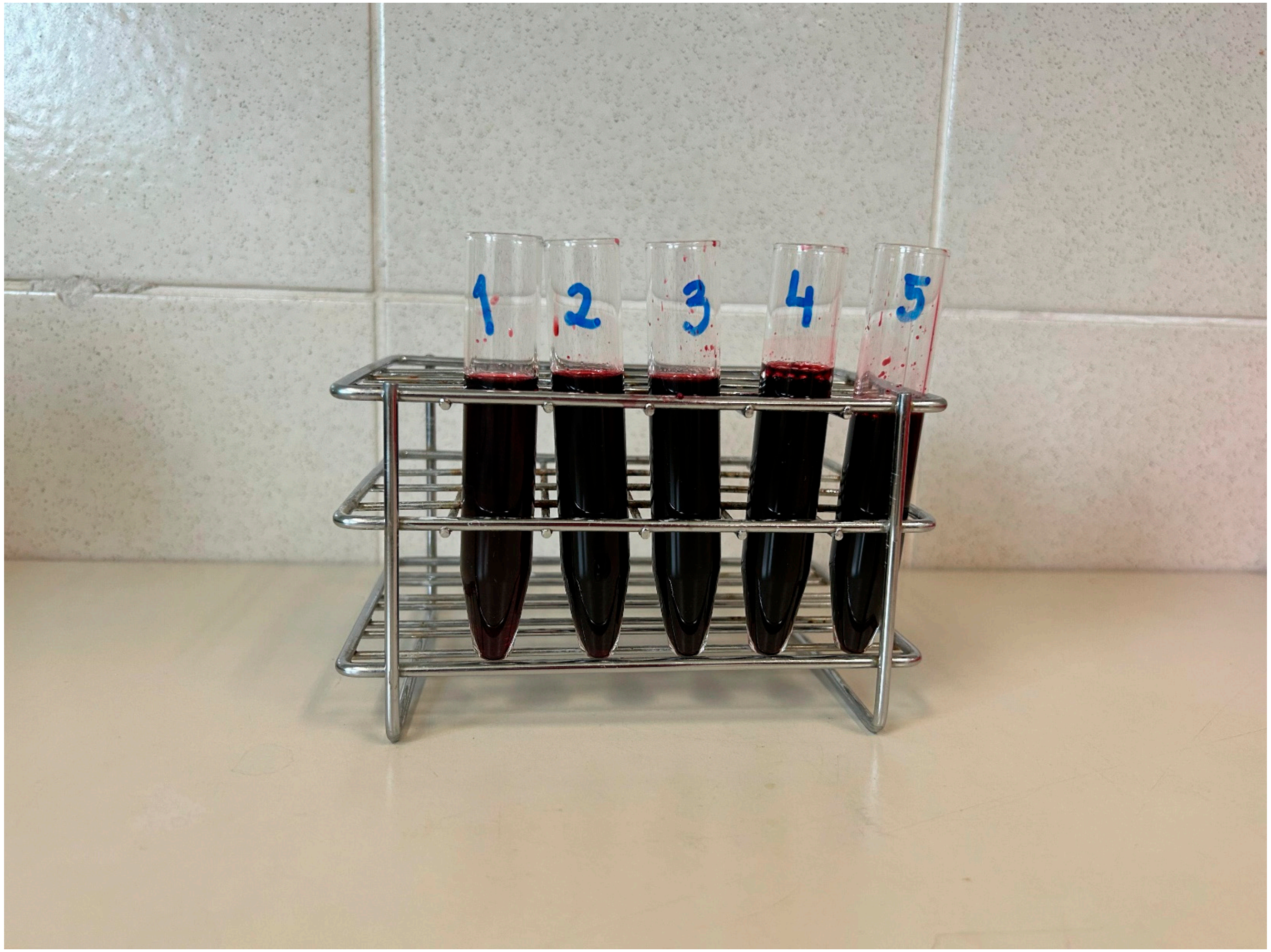

Figure S1: Photographs of the analyzed juices prepared by different maceration methods. 1- CM treatment; 2- TM treatment; 3- EM treatment; 4- US treatment; 5- MW treatment.

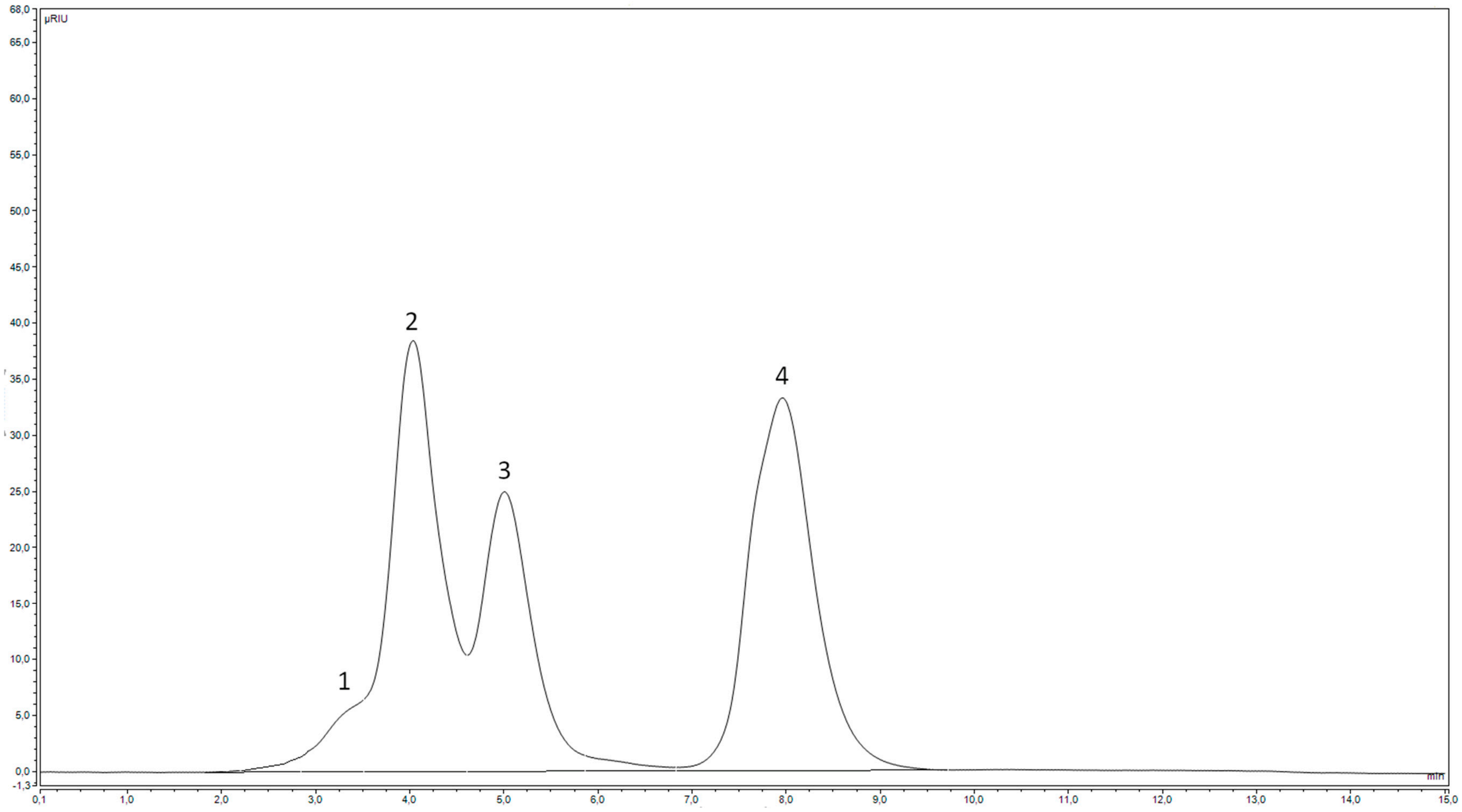

Figure S2: Chromatogram of detected individual sugars in aronia juice samples. 1- Sucrose, 2- Glucose, 3- Fructose, 4- Sorbitol.

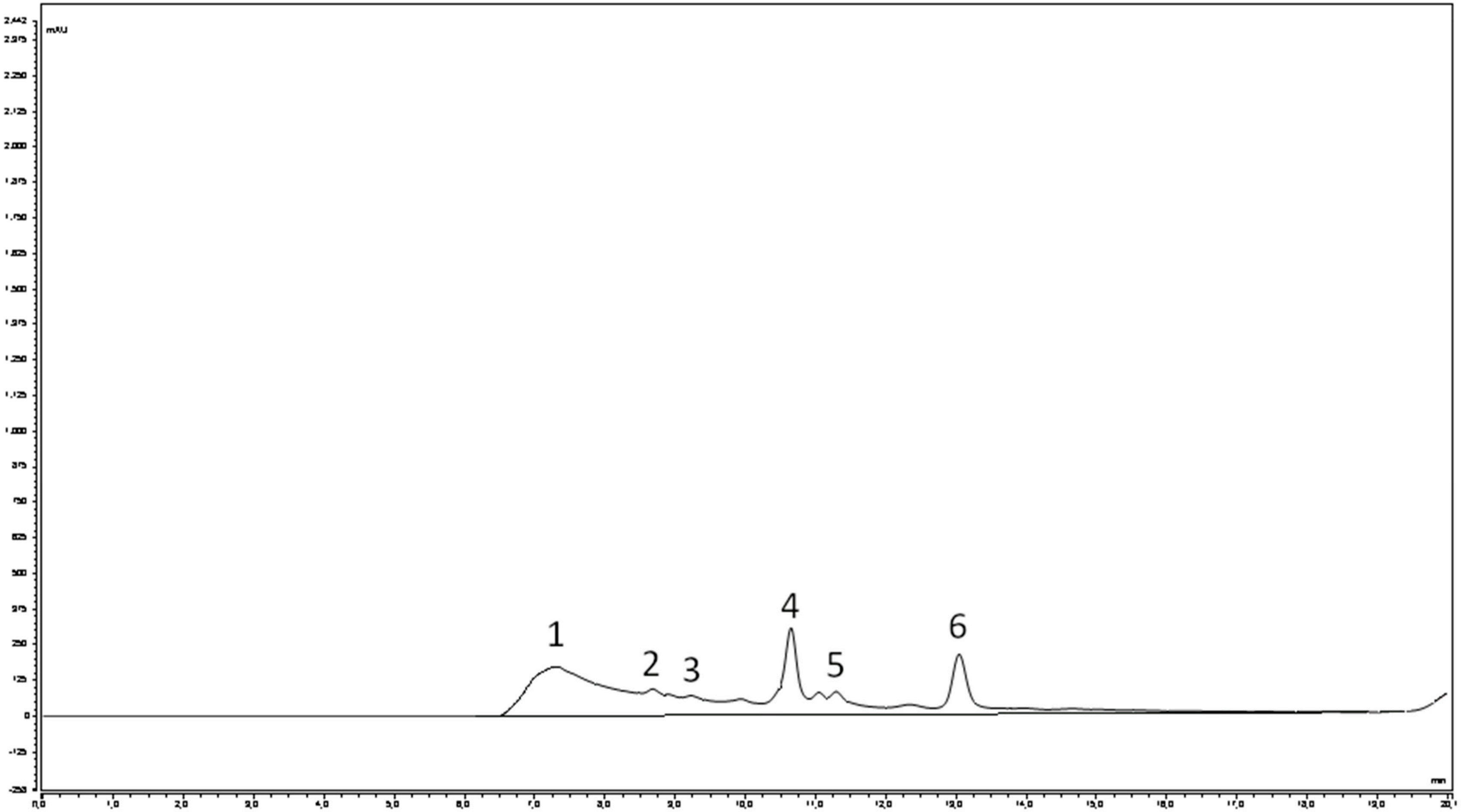

Figure S3: Chromatogram of detected individual acids in aronia juice samples. 1- Oxalic, 2- Citric, 3- Tartaric, 4- Malic, 5- Quinic, 6- Shikimic.

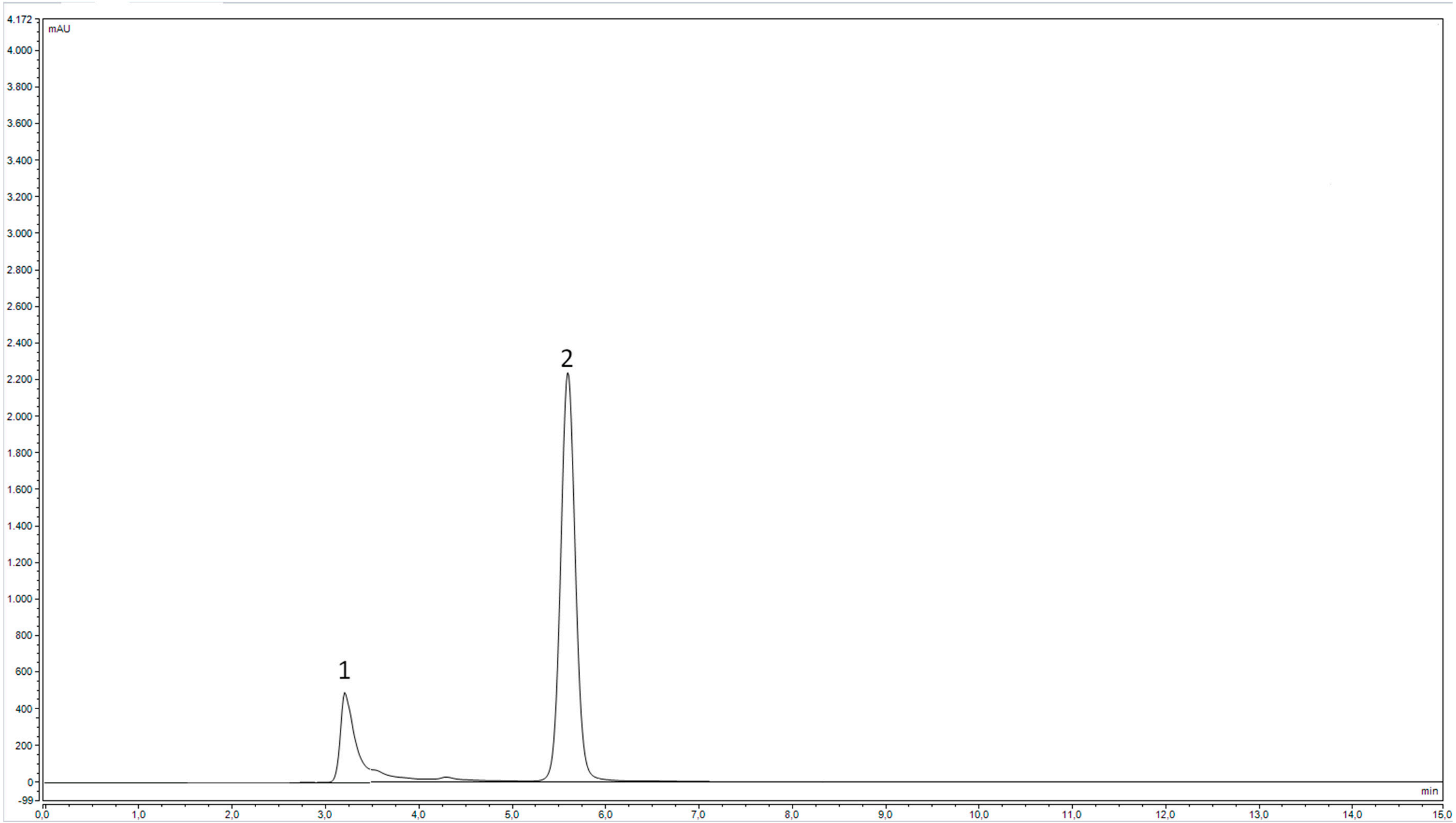

Figure S4: Chromatogram of detected D-ascorbic acid (1) and L-ascorbic acid (2).

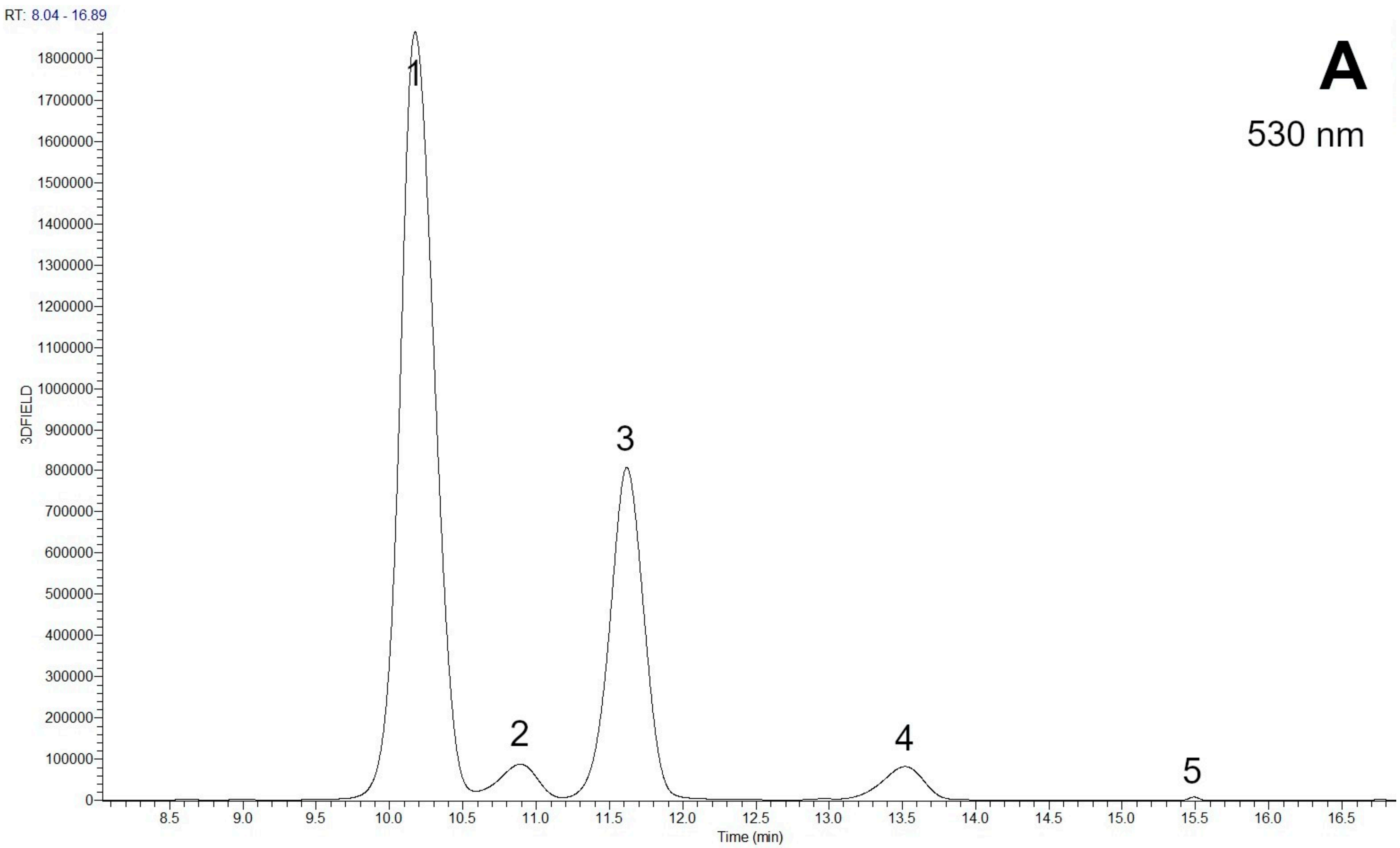
RT: 8.04 - 16.89
A
530 nm
3DFIELD
Time (min)
1
2
3
4
5

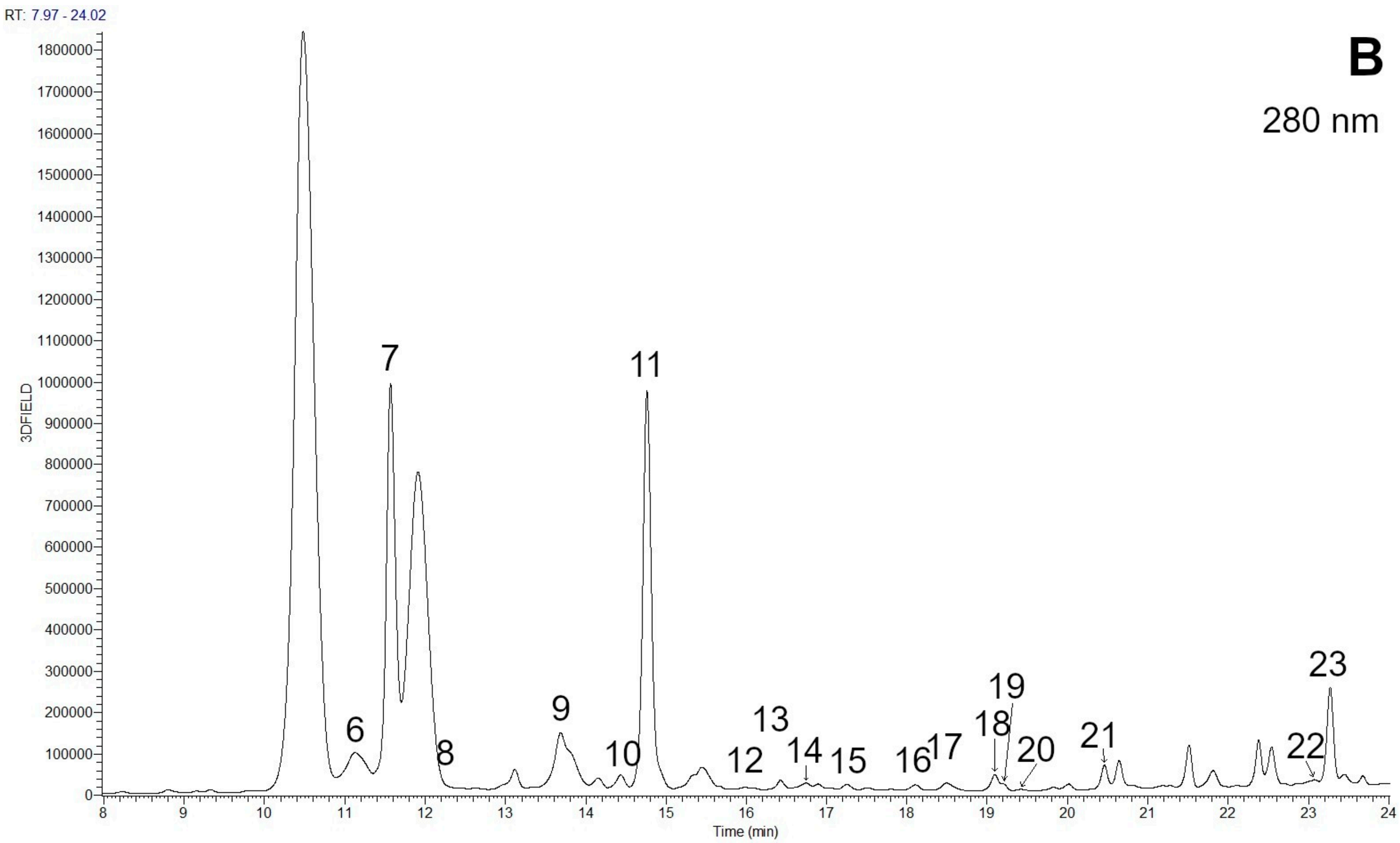
RT: 7.97 - 24.02
B
280 nm
3DFIELD
Time (min)
6
7
8
9
10
11
12
13
14
15
16
17
18
19
20
21
22
23

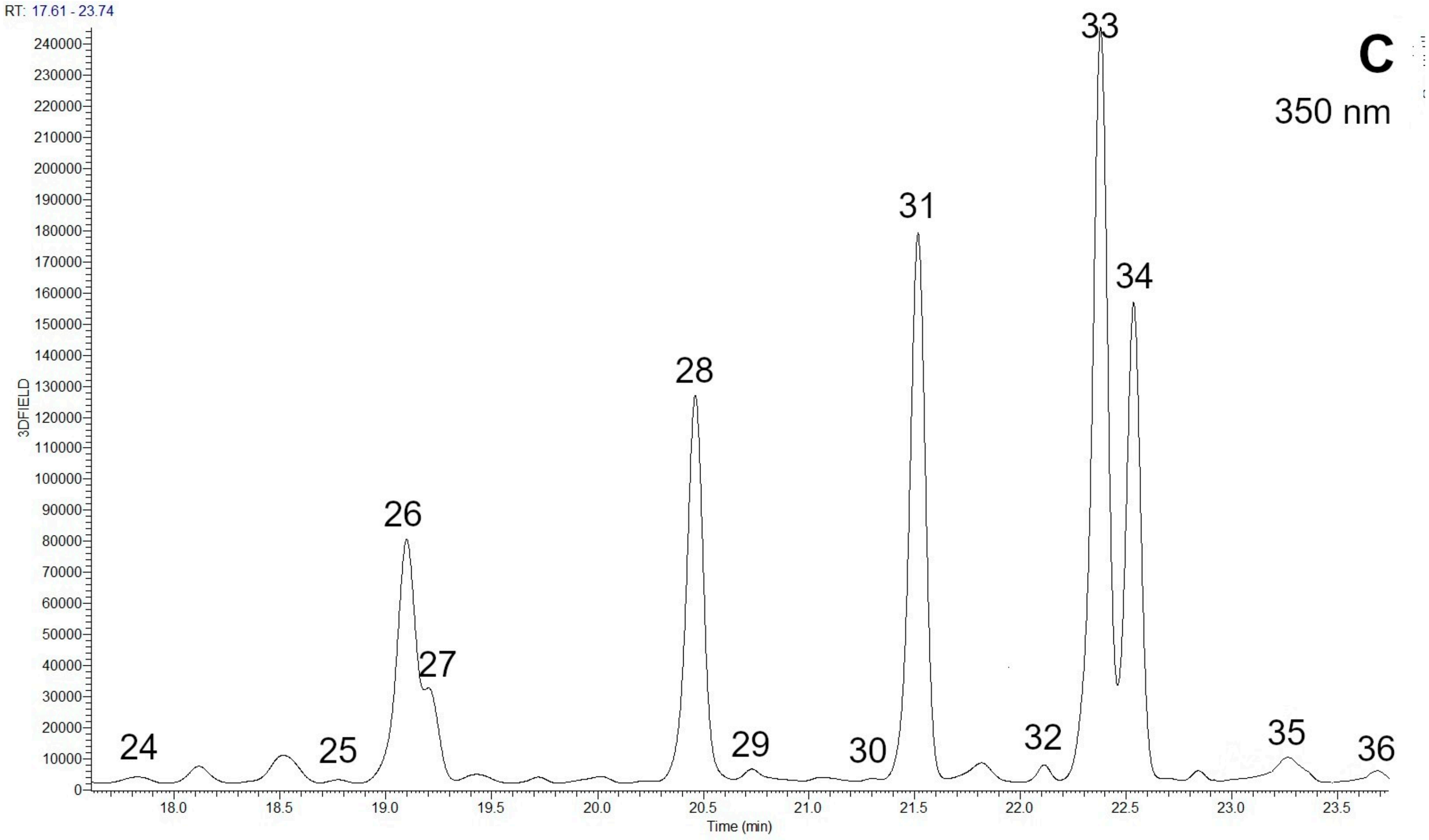

Figure S5: Chromatograms of detected individual phenolic compounds in aronia juice samples. **Chromatogram A:** 1 - Cyanidin-3-galactoside, 2- Cyanidin-3-glucoside, 3 - Cyanidin-3-arabinoside, 3 - Pelargonidin-3-galactoside, 4 - Cyanidin-3-xyloside, 5 - Cyanidin-3-O-hexosylpyruvate. **Chromatogram B:** 6 - Caffeic acid, 6 - Procyanidin hexamer 1, 7 - 3-caffeoylquinic acid, 8 - Procyanidin dimer 1, 9 - p-coumaric acid hexoside, 9 - Caffeic acid hexoside, 10 - 3-p-coumaroylquinic

acid, 11 - Procyanidin hexamer 2, 11 - 5-caffeoylquinic acid 1, 11 – Catechin, 12 - Procyanidin dimer 2, 13 - Procyanidin tetramer 1, 14 - 5-caffeoylquinic acid 2, 15 – Epicatechin, 16 - 4- Feruloylquinic acid, 17 - 5-p-coumaroylquinic acid 1, 17 - Procyanidin trimer, 18 - Procyanidin tetramer 2, 19 - Procyanidin dimer 3, 20 - 5-feruloylquinic acid, 21 - 5-p-coumaroylquinic acid 2, 22 - procyanidin tetramer 3, 23 - procyanidin tetramer 4. **Chromatogram C:** 24 - Apigenin dirhamnoside, 25 - Quercetin dirhamnosylhexoside, 26 - Quercetin dihexoside 1, 27 - Quercetin dihexoside 2, 28 - Quercetin-3-vicianoside, 29 - Procyanidin pentamer 1, 30 - Quercetin-3-robinobioside, 30 - Procyanidin pentamer 2, 31 - Quercetin-3-rutinoside, 32 - Naringenin hexoside 1, 33 - Quercetin-3-galactoside, 33 - Isorhamnetin pentosylhexoside, 34 - Quercetin-3-glucoside, 35 - Naringenin hexoside 2, 36 - Dicaffeoylquinic acid.
